# Impact of Training and Integration of Apps Into Dietetic Practice on Dietitians’ Self-Efficacy With Using Mobile Health Apps and Patient Satisfaction

**DOI:** 10.2196/12349

**Published:** 2019-03-04

**Authors:** Juliana Chen, Margaret Allman-Farinelli

**Affiliations:** 1 The University of Sydney, Charles Perkins Centre Discipline of Nutrition and Dietetics School of Life and Environmental Sciences Camperdown Australia

**Keywords:** dietetics, medical nutrition therapy, mHealth, patient satisfaction, smartphone

## Abstract

**Background:**

The use of mobile health (mHealth) apps in dietetic practice could support the delivery of nutrition care in medical nutrition therapy. However, apps are underutilized by dietitians in patient care.

**Objective:**

This study aimed to determine the feasibility of an intervention consisting of education, training, and integration of apps in improving dietitians’ perceived self-efficacy with using mHealth apps.

**Methods:**

Private practice Accredited Practising Dietitians who were not regular users or recommenders of mHealth apps were recruited into the intervention. The intervention consisted of 2 phases: (1) a workshop that incorporated an educational lecture and skill-building activities to target self-efficacy, capability, opportunity, and motivation factors and (2) a 12-week intervention phase allowing for the integration of an app into dietetic practice via an app platform. During the 12-week intervention phase, dietitians prescribed an Australian commercial nutrition app to new (intervention) patients receiving nutrition care. Existing (control) patients were also recruited to provide a measure of patient satisfaction before the apps were introduced. New patients completed their patient satisfaction surveys at the end of the 12 weeks. Usability feedback about the app and app platform was gathered from intervention patients and dietitians.

**Results:**

A total of 5 dietitians participated in the study. On the basis of an analysis of variance with the Tukey post hoc tests, the educational and skills training workshop component of the intervention produced immediate improvements in mean ratings for dietitians’ self-efficacy with using mHealth apps compared with baseline (*P*=.02), particularly with regard to *familiarity with apps* factor (*P*<.001). The self-efficacy factor *integration into dietetic work systems* achieved significant improvements from baseline to 12 weeks (*P*=.03). Patient satisfaction with dietetic services did not differ significantly between intervention (n=17) and control patients (n=13). Overall, dietitians and their patients indicated that they would continue using the app platform and app, respectively, and would recommend it to others. To improve usability, enhancing patient-dietitian communication mediums in the app platform and reducing the burden of entering in meals cooked at home should be considered.

**Conclusions:**

Administering an educational and skills training workshop in conjunction with integrating an app platform into dietetic practice was a feasible method for improving the self-efficacy of dietitians toward using mHealth apps. Further translational research will be required to determine how the broader dietetic profession responds to this intervention.

## Introduction

### Background

Mobile health (mHealth) apps targeting lifestyle-related behaviors, such as nutrition and exercise or fitness, are abundantly available in commercial app stores [1] and could be a potential medium for addressing the poor dietary and physical inactivity factors that are determinants of obesity and chronic diseases [2]. A previous review has outlined the areas in which dietitians can consider using apps to support their delivery of nutrition care in medical nutrition therapy [3], including streamlining of nutrition assessment, to maximize the time dietitians can spend on nutrition behavioral counseling [3]. Apps can also permit more timely and individualized patient-centered nutrition monitoring and evaluation and enable dietitian feedback [3].

Overall, 61.9% (353/570) of international dietitians report using mHealth apps in patient care and most commonly for patient education and self-monitoring of dietary behaviors [4], whereas use within the entire nutrition care process was less apparent [4,5]. The capability, opportunity, and motivations for dietitians in using mHealth apps in practice (behavior) were assessed using the behavioral system termed the Capability, Opportunity, Motivation-Behavior (COM-B) model [6]. In particular, this behavioral analysis identified that dietitians lacked both the capability and motivation to use apps [4]. Behavior change and performance of a behavior is also predicted by self-efficacy [7,8]. Perceived self-efficacy, defined as an individual’s beliefs about their capability to learn and perform particular behaviors [9], is considered to be an important precursor to the adoption of new technologies [10].

Dietitians indicated that, in part, their lack of capability and motivation and subsequent low self-efficacy toward using apps were because they were unfamiliar with the best apps to use and recommend and where apps could add value to nutrition care [4,11]. It was also found that the opportunity to use apps in practice was limited because of the lack of supportive physical infrastructure. According to the behavior change wheel, intervention functions are those that have the potential to address the deficits in the COM-B components [6]. Intervention functions identified as being able to increase dietitians’ app use behavior included education and skills training of dietitians and environmental restructuring, such as through the provision of physical app-based infrastructure [4].

Coaching and training workshops also enable individuals to gain mastery and proficiency in requisite skills, thereby increasing their self-efficacy toward new technologies [10]. Mastery experiences to build confidence in one’s abilities through successful performances are among the most effective influences on self-efficacy [7,9]. In addition, physical opportunities to engage in repeated practice of the behavior can facilitate mastery experiences [12]. Integration of mHealth apps into existing dietetic work systems may provide greater incentive for dietitians to adopt apps into their practice and build self-efficacy for their use [4,13].

Most platforms designed to support dietitians in managing their practice and patient records are software- or Web-based interfaces [14,15], including the Academy of Nutrition and Dietetics Health Informatics Infrastructure tool [16]. However, platforms with additional connectivity to apps are emerging. Such platforms can, for example, allow patients to obtain a brief automated nutrition assessment based on dietary guidelines and receive basic educational resources before being connected with a dietitian [17]. These platforms may also facilitate remote monitoring and evaluation of patient progress, allowing dietitians to send near real-time feedback on patient health behaviors via motivational messages [15,18-21]. Although many commercial diet-tracking nutrition apps exist, few have a platform for the exchange of data and for dietitians to view their patients’ app records [22]. The commercial Australian Easy Diet Diary app (Xyris Software Australia Pty Ltd, High Gate Hill, Australia) is an exception and links into a connected app platform that has been designed to support Australian dietitians in their provision of medical nutrition therapy to patients.

### Objectives

The aims of this study were twofold. The primary aim was to assess the feasibility of an intervention consisting of education and integration of apps into dietetic practice in improving dietitians’ perceived self-efficacy toward using mHealth apps in patient nutrition care. The secondary aim was to establish whether patient satisfaction would be enhanced following the integration of apps into dietetic services.

## Methods

### Study Design

Approval for this study was granted by the institutional human research ethics committee (approval number 2018/004). This was a pre-post study design involving 2 phases. In the first phase, dietitians attended an educational and training workshop with perceived self-efficacy with mHealth apps assessed before and after the workshop. The second phase of the study involved a 12-week trial. Dietitians were provided with the practical opportunity to use apps with their patients through a connected app platform that integrated apps into their dietetic practice. New patients who were counseled by these dietitians, hereafter referred to as *intervention patients*, were compared with existing patients who received dietetic consultation before the educational and training workshop, hereafter referred to as *control patients*.

### Recruitment and Participants

The study was advertised to dietitians via dietitian-specific electronic newsletters, websites, and social media pages as an educational and training workshop on how to enhance nutrition care through the incorporation of mHealth apps into practice. To be eligible, dietitians had to be (1) Accredited Practising Dietitians (APDs) working in the private practice setting (a minimum of 14 hours per week), (2) not regularly using or recommending mHealth apps in current patient care in dietetic practice (defined as using apps no more than 1-2 times per month), (3) not having used the Easy Diet Diary Connect platform (Xyris Software Australia Pty Ltd, High Gate Hill, Australia), and (4) willing to attend the in-person educational and skills training workshop in Sydney, Australia. Provisional APDs (ie, those in the first year of practice and still in a mentoring program) were excluded as they were deemed to have less practical dietetic experience and were more likely to have received education on the usage of apps at university. Dietitians were reimbursed Aus $200 for their time in participating in the study and recruiting patients. Dietitian participants were enrolled in February and March 2018.

Intervention patients recruited by their consulting dietitian were eligible to participate in the study if they (1) were 18 years or older; (2) had a health condition or chronic disease that would require self-monitoring of dietary intake; (3) were new patients; and (4) owned an iPhone, as the Easy Diet Diary app was only available on the iOS platform. Intervention patients with initial consultations with their dietitian between April and May 2018 were recruited into the study. Control patients had to be existing patients of the dietitian who had received at least one consultation with their dietitian before the study period but had not been receiving nutrition care for more than 6 months. Control patients were matched to intervention patients for gender and age range. These patients provided a retrospective measure of patient satisfaction with dietetic care before dietitians received the education and training and app platform. An Aus $10 shopping voucher was offered as an incentive to intervention and control patients following the completion of the patient satisfaction survey.

### Intervention

This intervention included 2 components: an educational and training workshop and a 12-week intervention phase where dietitians used the connected app platform. The intervention functions included in this study were designed to target the capability, opportunity, and motivation factors of the COM-B model [6] that previous research identified may facilitate increases in mHealth app uptake [4] (Table 1). Self-efficacy is also a predictor of behavior change [7,8]. Therefore, the intervention also addressed all 4 sources of influence on self-efficacy—mastery experiences, vicarious experience, social persuasion, and somatic and emotional states [7,9]—to improve dietitians’ beliefs in their capability to use apps in their practice.

#### Educational and Skills Training Workshop

All eligible dietitians were required to attend the face-to-face 4-hour educational and skills training workshop held on a weekday during business hours in Sydney, Australia. At the workshop, dietitians were provided with education on how a range of apps (eg, Easy Diet Diary, Noom Coach by Noom Inc., New York, US, and FoodSwitch by The George Institute for Global Health, Sydney, Australia) could be used at each step of the nutrition care process to support patient nutrition care based on the most current evidence [3], and case study activities were used to apply this knowledge and build mastery of skills. To overcome a key psychological capability barrier for dietitians around the lack of awareness of the best apps to use in dietetic practice [4], the workshop also educated dietitians about the range of commercially available mHealth apps. Dietitians were trained to appraise and evaluate the quality of these nutrition apps.

Practical and interactive opportunities familiarized dietitians with how to download and navigate through common functions of diet-tracking nutrition apps not only to gain further mastery experiences but also to enhance psychological capability. Support and modeling were provided by the workshop facilitator (JC) and other participating dietitians. Finally, dietitians were trained in the use of the commercial app platform (Easy Diet Diary Connect). Relevant patient tools and information resources were created, including instructions on how to download, install, and use the companion app Easy Diet Diary.

#### 12-Week Intervention Phase

In the 12 weeks following the workshop, dietitians were instructed to provide standard nutrition counseling and care. For the intervention patients, dietitians were also to prescribe the Easy Diet Diary app as a dietary record for dietary assessment and self-monitoring and to review these app records via the Easy Diet Diary Connect platform. Control patients were not prescribed any apps. Enablement and physical opportunities to enhance dietitians’ self-efficacy for using mHealth apps were provided through the app platform. Researcher support was made available during this period for any difficulties encountered with the app or app platform.

#### Easy Diet Diary and Easy Diet Diary Connect Platform

**Figure 1:**
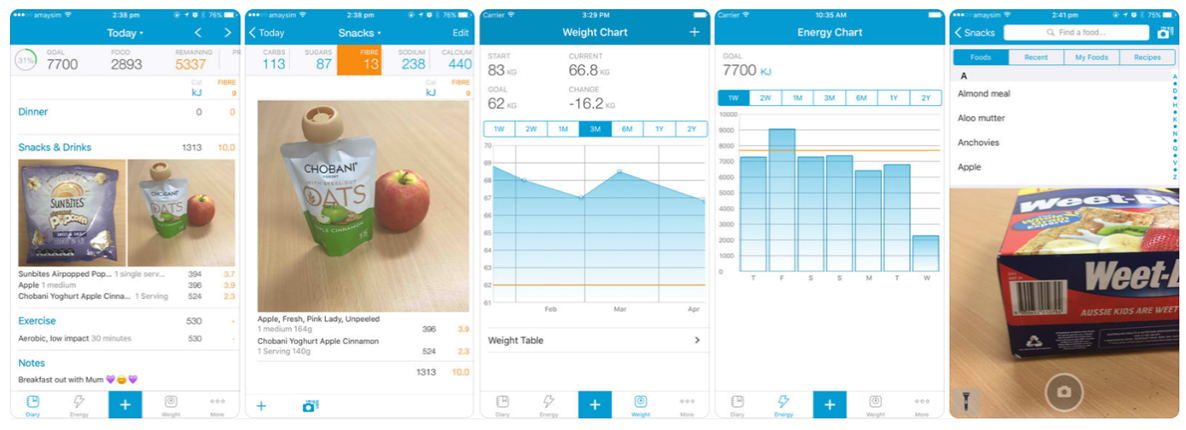
Screenshots of the Easy Diet Diary app (Xyris Software Australia Pty Ltd).

As dietitians prefer country-specific food databases [13], the Easy Diet Diary [23] app (Figure 1) was selected for implementation as the app primarily draws upon the Australian Food and Nutrient Database AUSNUT 2011-2012, which contains foods specific to the Australian food supply. The relative validity of the energy and macronutrient output from the app when compared with 24-hour recalls has been previously established [24]. Unique to the app is also its ability for users to send data directly to their dietitian, which can be analyzed further and used in dietary assessment via access through the FoodWorks nutrient analysis software (Xyris Software Australia Pty Ltd, High Gate Hill, Australia) [25].

**Figure 2:**
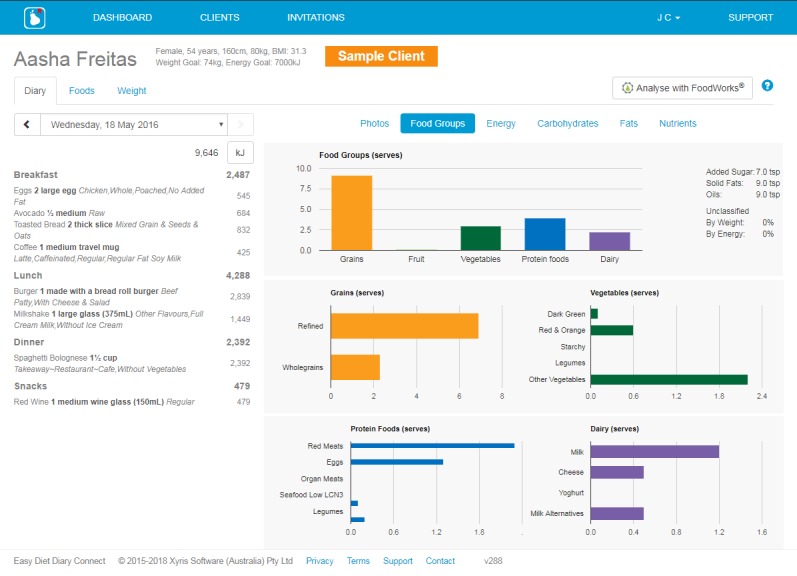
Screenshot of the Easy Diet Diary Connect platform (Xyris Software Australia Pty Ltd).

The release of a secure Web browser–based interface, the Easy Diet Diary Connect platform [26] (Figure 2), allows dietitians to view their patient’s dietary records from the Easy Diet Diary app in real time. The Easy Diet Diary Connect platform makes patient food records and energy and macronutrient intake breakdowns for each day available to the dietitian for reviewing. The interface also automatically displays in a chart format a qualitative analysis of dietary intake based on food groups compared against recommended serves from dietary guidelines. Charts to monitor self-reported weight are also accessible.

**Table 1:** Intervention functions included in the education and training workshop and 12-week intervention phase that targeted deficits in the sources of behavior (capability, opportunity, and motivation) as classified by the Capability, Opportunity, Motivation-Behavior (COM-B) model, and the sources of self-efficacy, according to Bandura.

Intervention functions	Sources of behavior targeted	Sources of self-efficacy targeted
Education-component of workshop: education to impart knowledge, awareness, and instructions about how apps could be used to support the nutrition care process and dietetic services; what the best apps to recommend to patients are; and the limitations of apps particularly with regard to their quality and accuracy of commercial mHealth apps.	Psychological capability, reflective motivation, and automatic motivation	Social or verbal persuasion
*Training-component of workshop*: training to provide opportunity to behavioral practice, develop and master skills with using apps, and achieve personal performance accomplishments, particularly through case study activities to apply apps across the nutrition care process; appraisal and evaluation of app quality; hands-on experience with downloading/accessing, using and navigating through different functionalities of apps, including Easy Diet Diary and Easy Diet Diary Connect platform. *12-week intervention phase*: enablement by environmental restructuring through the provision of the Easy Diet Diary Connect platform to integrate patient use of the app into dietetic practice and continued practice with reviewing patient app records via Easy Diet Diary Connect platform	Physical and psychological capability and physical opportunity	Mastery experiences
Expert and credible workshop facilitator who is a dietitian, modeling and demonstrating competent use of apps and platform, participant modeling of successes in using apps, working in small groups during workshop activities when using apps, and platform to allow participants to observe others similar to them for social comparison, social support, and successful accomplishment in using apps	Social opportunity	Vicarious experience
Workshop facilitator provision of supportive feedback on participants’ behavior and performance to enable them to refine their skills with using apps; persuasion and exhortation of participants that they have the capability to master app use even in difficult situations, such as short consultations, to give dietitians provisional self-efficacy and the belief and support for attempting the behavior; encouragement provided by workshop facilitator and other participating members; and ongoing workshop facilitator support with app/app platform use during the 12-week intervention phase for enablement	Social opportunity, reflective motivation, and automatic motivation	Social or verbal persuasion
Positive and encouraging workshop environment, with minimization of situations that arouse stress and anxiety; continued and regular prescription of Easy Diet Diary to patients, so that use becomes easy and habitual in dietetic practice	Automatic motivation	Somatic and emotional states

### Outcome Measures

#### Primary Outcome

The primary outcome variable of this study was the change in ratings for dietitians’ self-efficacy with using mHealth apps. Self-efficacy was measured via Web-based surveys at 3 time points: at baseline (1 week before attending the workshop), after the educational and skills training workshop (post workshop), and at the end of the 12-week intervention period. A 16-item validated survey tool for measuring self-efficacy with using mHealth apps among dietitians was used [27].

#### Secondary Outcomes

The secondary outcome was the impact of the intervention on patient satisfaction with the dietetic services and nutrition care provided assessed using a satisfaction questionnaire adapted from a previously validated tool designed for outpatient dietetic services [28]. The questions relating to written information were altered to relate to general tools used in dietetic practice, so as to also capture the impact of the mHealth app on patient satisfaction.

Control patients completed the survey at the beginning of the 12-week intervention period to provide a measure of patient satisfaction with dietetic care before the apps were introduced, and intervention patients completed it at the end of the 12-week intervention. Control and intervention patients were also asked to provide details regarding whether they had used mHealth apps before coming to see their dietitian. Both groups recorded their age and gender in the survey (as age may determine how *savvy* they are with technology).

Additional outcome measures collected from dietitians included personal app use and recommendation of apps in patient care, derived from a previously piloted and validated survey administered to dietitians [4]. Other basic demographic questions including age, gender, and length of dietetic practice were also collected.

#### Process Outcomes

Process evaluation for the intervention was conducted to provide feedback to inform future larger-scale dissemination. To evaluate the training workshop, dietitians completed a Web-based questionnaire after the workshop regarding their satisfaction with the workshop content and delivery style and provided free-text comments on suggestions for future modification and improvement of the workshop. At the end of the 12-week intervention period, dietitians were asked to indicate their practices around reviewing app records.

The validated 10-item System Usability Scale (SUS) survey, with minor modifications whereby the term *system* was substituted for *app* *platform* or *apps*, was used to collect an assessment of system usability [29,30]. Additional questions based on an acceptability questionnaire for a mobile diabetes management system, WellDoc [31], were included in the 12-week dietitians survey to understand whether the app platform was helpful to them and their patients in terms of their relationship and the additional care and the acceptability of the amount of time spent performing tasks on the app platform. Open-ended questions were also included to gather any feedback from dietitians and patients regarding the usability and any suggested improvements to the features and functionality of the connected app platform and app, respectively.

### Data Analysis

A mixed-methods approach was used for data analysis. Descriptive statistics were generated for quantitative measures such as participant demographics and outcome variables (mHealth app self-efficacy and patient satisfaction). Analysis of variance (ANOVA) followed by the Tukey post hoc tests was conducted to determine any differences in the mean changes in items measuring dietitians’ self-efficacy with using mHealth apps between time points. Logistic regression models were conducted to assess any differences between patient satisfaction ratings (dependent variable) from intervention and control patients (independent variables), adjusting for other covariates including the dietitian that patients saw, patient age, gender, and previous experience with using mHealth apps. All statistical analyses were performed using SPSS statistical software, version 22.0 [32]. The SUS scores were calculated based on the original method described by Brooke [29], with scores above 70 considered to reflect acceptable usability [30,33]. Qualitative inductive thematic analysis was used to code open-ended responses into themes.

## Results

### Participant Characteristics

Overall, 31 dietitians responded to the screener survey, of which 2 were partial responses and 22 were excluded based on the eligibility criteria. Of the 7 eligible dietitians, 2 were unable to attend the workshop, but the other 5 dietitians attended and completed the 12-week intervention. All the participants were female and aged between 46 and 65 years and with over 20 years of experience in practicing. The most common practice areas were in weight management (n=4) followed by diabetes (n=3).

Of the 5 dietitians, 4 had personally used apps and 3 had personally used health apps. As per the inclusion criteria, all participating dietitians did not frequently use apps in their practice (1-2 times per month or less), all citing that a lack of awareness about the best app to use was a key barrier. Other barriers included a lack of time to discuss apps in a consultation (n=2), lack of infrastructure (eg, no access to Wi-Fi; n=2), topics covered by apps not relevant to clientele (n=1), and apps being too hard to use (n=1).

They had all previously recommended apps to their patients, 1 to 2 times per month (n=3) or 1 to 2 times per year (n=2). On average, they had recommended 3.4 apps (SD 0.9) over the past year to patients, with the Low FODMAP Diet informational app by Monash University, Melbourne, Australia recommended by all, and 2 dietitians having recommended Easy Diet Diary previously.

Furthermore, 13 of 19 control patients who attempted the patient satisfaction survey completed it. In addition, 23 intervention patients provided consent to being issued the survey at the end of the 12-week intervention, with 17 patients completing the survey. Intervention and control patient demographics are outlined in Table 2. The majority of patients had not used an mHealth app before coming to see their dietitian and none had previously used the Easy Diet Diary app.

### Impact of Intervention on Outcomes

#### Dietitians’ Self-Efficacy With Using Mobile Health Apps

On the basis of the mean overall ratings for the dietitians who participated in the intervention, there was a significant improvement in overall self-efficacy with using mHealth apps (ANOVA *F*_2,12_=7.0; *P*=.01). The Tukey post hoc test revealed significantly higher postworkshop mHealth app self-efficacy ratings compared with baseline (*P*=.02), which were sustained at 12 weeks (*P*=.01; see Table 3).

**Table 2:** Demographics of intervention (n=17) and control patients (n=13) who completed the survey (N=30).

Characteristics		Intervention patients, n	Control patients, n
**Age (years)**			
	18-30	2	2
	31-40	7	5
	41-50	1	3
	51-60	2	0
	More than 60	5	3
**Gender**			
	Female	14	13
	Male	3	0
**Use of a mobile health app before coming to see their dietitian**			
	Yes	5	4
	No	12	9

**Table 3:** Dietitians’ self-efficacy with mobile health apps before and after attending the educational and skills training workshop on apps as well as after 12-weeks of practical opportunities to use mobile health apps in their practice. The mean ratings for individual items and factors are presented. One-way analysis of variance was conducted followed by the Tukey post hoc test.

Self-efficacy item^a^		Baseline rating	Post workshop		End of 12 weeks	
			Rating	*P* value	Rating	*P* value
**Familiarity with apps factor**		5.8	8.9^b^	.001	8.8^b,c^	.001
	When I currently recommend/in the past have recommended mobile health apps to patients	4.6	8.2^b^	.005	8.4^b,c^	.004
	When I am familiar with which mobile health apps to recommend	7.0	9.6^b^	.07	9.2^b,c^	.02
**Training and support factor**		7.5	9.3^b^	.01	8.6^c,d^	NS^e^
	When someone else has helped me get started	8.0	9.8^d^	NS	8.6^c,d^	NS
	When I can call someone for help when I get stuck	8.6	10.0^d^	NS	8.6^c,d^	NS
	When there is no one around to tell me how to use them as I go	4.8	7.8^d^	NS	7.8^c,d^	NS
	When someone has shown me how to use them first	8.6	9.6^d^	NS	9.4^c,d^	NS
**Efficiency and effectiveness of nutrition care factor**		6.6	7.6^d^	NS	8.1^c,d^	NS
	To improve the efficiency of consultations	7.2	7.4^d^	NS	7.6^c,d^	NS
	To improve the effectiveness of nutrition interventions	7.2	8.2^d^	NS	8.6^c.d^	NS
	To improve patient health outcomes	7.0	8.4^d^	NS	9.0^c,d^	NS
	When I need to deliver nutrition interventions	7.2	8.0^d^	NS	9.2^c,d^	NS
	When I need to conduct nutrition assessments	7.0	8.2^d^	NS	8.8^c,d^	NS
	When there is a short consultation time	5.0	5.2^d^	NS	5.2^c,d^	NS
	When patients ask me about using mobile health apps	5.4	7.8^b^	0.03	8.6^b,c^	0.005
**Integration into dietetic work systems factor**		7.0	8.1^d^	NS	8.8^b,c^	0.03
	When apps are integrated into my existing patient management systems	6.2	7.2^d^	NS	7.8^c,d^	NS
	When there is an app platform where I can view patient mobile health app records/data	6.8	8.0^d^	NS	8.8^c,d^	NS
	When I want patients to self-monitor their behaviors	8.0	9.2^b^	.001	9.8^c,d^	.001
Mean overall rating		108.6	132.4^b^	.02	135.4^b,c^	.01

^a^Dietitians rated each item from 0 indicating *I am not able at all*, to 5 indicating *Moderately certain I am able*, and to 10 indicating *Completely certain I am able*.

^b^Significant difference from baseline.

^c^Nonsignificant difference from post workshop.

^d^Nonsignificant difference from baseline.

^e^NS=nonsignificant *P*>.05.

Attendance at the educational and skills training workshop significantly improved dietitians’ familiarity with apps (*F*_2,12_=21.2; *P*<.001) from baseline to the postworkshop (*P*<.001) and 12-week measures (*P*<.001) as well as across all items that this self-efficacy factor was comprised. Significant improvements from baseline were also observed in the postworkshop self-efficacy ratings for the training and support factor but not in the subitems. There were no statistically significant differences between postworkshop and 12-week ratings for this factor or for the other 3 factors, individual mHealth app self-efficacy items, or mean overall self-efficacy.

Despite significant improvements in the efficiency and effectiveness of nutrition care factor subitem regarding patient queries with using mHealth apps, 1-way ANOVA revealed no significant differences between any of the 3 time-point measurements for this factor (*F*_2,12_=2.3; *P*=.1). With the implementation of the connected app platform in the 12-week phase, ratings for the integration of apps into dietetic work systems factor significantly improved from baseline to 12-week measures (*P*=.03). The factor subitem regarding patient self-monitoring of health behaviors significantly improved post workshop (*P*=.001), with self-efficacy maintained at 12 weeks (*P*<.001).

#### Patient Satisfaction

Table 4 presents the mean patient satisfaction ratings for intervention and control patients based on each item as well as for the 4 factors of the tool, including perceived health benefits, staff presentation and interpersonal skill, fulfilled expectations, tools and materials, and overall satisfaction. Both intervention and control patients agreed or strongly agreed with the majority of satisfaction items and rated the overall satisfaction with the dietetic services they received as good to very good.

Logistic regression showed no difference in patient satisfaction with dietetic services between intervention and control patients, when adjusting for their dietitian (χ^2^_2_=1.8; *P*=.4). Additional adjustment for patient age, gender, and experience with mHealth apps did not change this finding (χ^2^_5_=6.0; *P*=.3).

**Table 4:** Patient satisfaction ratings with dietetic services.

Patient satisfaction item^a^		Intervention patients (rating from 1 to 5)	Control patients (rating from 1 to 5)
**Perceived health benefits**		3.9	4
	The care I received from the dietitian has improved my general health	4	4.2
	The care I received from the dietitian has improved the results of my medical treatment	3.6	4
	The care I received from the dietitian has helped me achieve my health goals	3.9	3.8
	The care I received from the dietitian has helped me to feel healthier	4.2	4.1
**Staff presentation and interpersonal skill**		4.6	4.6
	The dietitian listened carefully to what I had to say	4.6	4.6
	The dietitian was attentive to my needs	4.6	4.5
	The dietitian came up with a good plan for helping me	4.5	4.5
	The dietitian was well presented	4.6	4.7
	The dietitian was polite and courteous	4.7	4.7
	The dietitian was friendly	4.8	4.8
**Fulfilled expectations**		4.5	4.6
	The nutrition care I received was helpful	4.4	4.5
	The nutrition care I received met my expectations	4.2	4.4
	I would recommend the nutrition service provided by my dietitian to other members of the community	4.7	4.8
**Tools and materials**		4.4	4.3
	The tools were of a high standard	4.4	4.1
	I found the tools very easy to understand	4.4	4.3
	The tools were easy to use	4.3	4.3
	The tools made sense	4.4	4.3
	The tools were well presented	4.3	4.3
Overall, how would you rate your satisfaction with the services provided by your dietitian?^b^		4.8	4.5

^a^Patients rated items 1 to 18 from 1=strongly disagree to 5=strongly agree.

^b^Patients rated item from 1=very poor to 5=very good.

**Table 1:** Dietitian satisfaction with the educational and skills training workshop component of the intervention.

Items	Rating^a^, mean (SD)
The theory and practical components of the workshop improved my understanding of the topics covered	4.4 (0.55)
This workshop helped me develop skills applicable to my professional practice	4.2 (0.45)
I can see how the knowledge and skills I am learning can be put to use in my future professional work	4.2 (0.45)
I have come to feel more confident about my ability to use apps in my dietetic practice and in patient nutrition care	4.4 (0.89)
Feedback provided during the workshop was helpful to my learning	4.6 (0.55)
Overall, I was satisfied with the quality of this workshop	4.6 (0.55)

^a^Dietitians rated each item from 1=strongly disagree to 5=strongly agree.

### Process Evaluation

Overall, dietitians agreed or strongly agreed that they were satisfied with the quality of the workshop (Table 5). All dietitians who attended indicated that they would recommend this workshop to colleagues, with 1 participant relaying how she “thoroughly enjoyed the workshop and learned a lot.” Dietitians’ favorite parts of the workshop included the *practical elements* that provided them with hands-on experience with using apps as well as the demonstrations by the workshop facilitator on how to use the connected app platform: *Being shown how to use the Easy Diet Diary platform*. Activities and information to raise awareness about the quality and range of different apps available to use in patient nutrition care and dietetic practice were also cited as a liked aspect of the workshop: *Learning pros and cons about various apps*. The workshop helped to transform dietitians’ psychological states and motivations toward using apps, with 1 dietitian describing how the workshop “Made me feel positive towards integrating apps in my work” or “I’m looking forward to integrating Easy Diet Diary into my practice.”

For improvement of the workshop, feedback revolved mainly around timing, such as breaking up the workshop to allow for breaks and reconsidering the length of the workshop. The educational lecture component of the workshop was recognized to be important and interesting. However, suggestions were made to transfer some of the time allocated for knowledge exchange to even more skill-based training and opportunity for gaining practical experience with apps. For example, 1 dietitian mentioned:

While interesting the theoretical background was a little long.

Another dietitian stated:

Needed more time with the hands-on. Would have like[d] to play with a variety of apps rather than just slides [learning about them].

Drawing further upon the social support of colleagues, it was also expressed that the workshop could be improved by allowing for more mastery of skills:

It may have been useful to practise via role play.

#### Dietitians’ Reviewing of App Data

Before participation in this intervention, 4 of the 5 dietitians reviewed their patients’ progress with the mHealth apps recommended and 1 never did—predominantly talking about the progress made with the app without looking at the data (n=3), the other saw the data as to:

...just provide back up information rather than tracking.

For all dietitians, a key barrier to reviewing patient app records involved inadequate knowledge, experience, or confidence regarding which apps would allow for reliable data sharing. Furthermore, dietitians perceived that there was a lack of time in the actual appointment for reviewing the records.

Following the intervention, dietitians reviewed their patient app records more frequently than at baseline, with 3 of the 5 dietitians reviewing app data in some consultations—without reference to the data, or through reviewing data on their patient’s smartphone, or via the connected app platform. In addition, 1 dietitian reviewed their patient app records via the connected app platform on their computer at every consultation, and another dietitian reviewed records through the platform even between consultations and subsequently provided encouragement emails to her patients.

For these 2 dietitians who reviewed their patients regularly (ie, at every consultation or in between consultation), they strongly agreed that the connected app platform had been helpful to their practice, whereas those reviewing patient app records less frequently agreed or were neutral. They also agreed that their relationship with their patients has improved because of this app platform system, whereas those who did not review regularly provided a neutral response. The majority agreed or strongly agreed (4/5) that their patients had found the integrated app platform to be helpful in addition to usual dietetic care.

#### Easy Diet Diary Connect Usability and Acceptability

Dietitians rated the usability of the Easy Diet Diary Connect platform with a mean rating of 73 (range: 57.5-100). All dietitians indicated they would continue to use the app platform and recommend it to others. Qualitative feedback from dietitians revealed that the connected app platform was easy to use and a good tool in patient care for “Tracking of food and nutrient intakes.” Dietitians found that the app platform provided an additional source of information to assist with the tailoring of nutrition interventions:

It gave me the information I needed to modify the patient's education, in a clear concise form

Additional features that were suggested to improve the platform included the “option for more nutrients.” Dietitians also wanted further communication mediums to be integrated within the connected app platform, for example, the “Ability to send patients SMS messages from platform.” However, dietitians did note the varied responses in patient willingness to share their health data:

A couple of them were too uncomfortable to link me in (they were in the older age group).

In addition, 1 dietitian encountered technical issues, citing that “being unable to access my [connected app platform] account made it very difficult,” to review patient records in a timely manner; this created a loss in momentum and motivation to continue using the app platform.

When used in conjunction with the app platform, dietitians believed that Easy Diet Diary was a “great app but just don’t have the time to deliver and monitor—I will recommend this app for people to self monitor.” Time constraints around teaching patients how to use the app were raised, with particular consideration around patients’ own self-efficacy with using mHealth apps:

As I have a structured consultation, I found it difficult to include the app introduction and downloading in the consultation, therefore they were asked to download in their own time, which they often didn't. It was also very time consuming for me to monitor patients and give feedback between consultations, it really depends on each patient re understanding and motivation with technology.

There were mixed responses from dietitians toward the acceptability of the amount of time spent performing tasks on the app platform per patient. Overall, 1 dietitian disagreed and 3 were neutral, whereas the dietitian who reviewed apps in every consult agreed that the time spent was acceptable. On average, dietitians spent 13 min (range 5-15 min) per patient on app platform–related activities (eg, teaching patients how to download and set up the app and viewing their app records). The majority (4/5) of dietitians agreed that the app platform helped to improve the amount of time they spent on dietary assessment.

#### Easy Diet Diary App Usability From Patients’ Perspectives

A mean SUS rating of 77 (range: 55-100) for the Easy Diet Diary app was indicated by patients. All except 1 patient would continue using the app. This patient cited that the reason for not continuing use was because the app was:

Not particularly compatible with alternative nutrition approaches e.g. Keto/paleo/5:2.

r5, female, 31-40 years old

All patients reported they would recommend the app to others.

The key themes emerging from patient feedback about the aspects they liked about the app included that it was easy to use. Functionalities within the app identified as enhancing the ease of use were related to the logging of dietary intake. These included the copy and paste functionality, “Easy to copy and paste daily meals to other days. e.g. if you have the same breakfast everyday” [r3, female, >60 years old], barcode scanner, “barcode scanner was excellent” [r5, female, 31-40 years old], and recent function, “(took me a little time to identify that one)... Saved me much time in keeping my records up to date” [r15, male, 51-60 years old].

Patients found the app useful for tracking calories and nutrients:

Counting the calories & seeing how much protein & calcium in my diet.

r10, female, 31-40 years old

The feature to highlight selected nutrients of interest in the app was liked:

The days total and the option to choose what one is of most importance to me to quick tally in the orange writing.

r16, female, 18-30 years old

Some patients made comparisons between the Easy Diet Diary app and other nondietitian-designed commercial nutrition apps:

It’s also not as pushy as MyFitnessPal. [r1, female, 31-40 years old].

However, there was not necessarily an understanding of how the Easy Diet Diary app was different or better compared with other apps:

Although it does seem similar to others out there, such as My Fitness Pal [sic]. I’m not sure what would differentiate it from the rest. But useful nevertheless.

r12, female, 31-40 years old

One patient relayed how the app had been supportive in helping her achieve her health goals and improving her health outcomes:

The ap[p] has helped me to lose weight and over time help me with my diabetes- am aiming to remove the meds all together.

r15, male, 51-60 years old

Another patient reflected upon needing to be more adherent to using the app to self-monitor their dietary intake:

I need to be disciplined and complete my daily diet intake every day

r7, female, >60 years old

The notes section of the app was highlighted as facilitating communication and accountability between the patient and their dietitian and, thus, also creating a sustained interest to continue using the app. Of the patient, 1 described how:

I was happy to be using it with my dietician [sic], knowing that they would and could be checking what I had been logging regularly. It kept me more accountable.

r12, female, 31-40 years old

Nevertheless, it was perceived that the app could offer more tailored features to complement their dietitians’ approach and nutrition care goals:

Tailored target setting based on nutrition approach e.g. Less emphasis on calories, good fats vs bad fats etc.

r5, female, 31-40 years old

Opinions on the Easy Diet Diary app food database varied between patients. Some patients identified the database as being an aspect they liked: “I also liked the preloaded nutritional value of products that I can buy from the shops” [r16, female, 18-30 years old], particularly also given that the app used an Australian database of foods:

It’s [the app is] Australian so has a lot of Australian products and foods included.

r1, female, 31-40 years old

However, this same participant also expressed challenges with being unable to find certain food options. She suggested that the database should contain more generic food items and less supermarket brands, especially for common food items such as bread and milk:

There’s not a generic “sourdough” you have to choose a random supermarket brand which may or may not be similar.

For the low-fat (light blue) milk option, it forces you to choose one that’s omega enriched. Why isn’t there just a standard option for this?

To increase the relevance of the database, regular updating was suggested: “Keeping up to date more items” [r11, female, 31-40 years old], as well as refining the database of foods to make searching of foods easier and quicker:

Many products are in the app, however, are not easy to find. Suggest that a food type heading be add[ed].

r9, male, >60 years old

Others wanted the types of food available in the database to be expanded, for example, to include fast food options:

Getting many of the take away type foods into the ap[p]. Eg Red Rooster Tropical Pack.

r15, male, 51-60 years old

For patients who cooked meals at home themselves, they reported it as being burdensome to enter all the ingredients to these meals:

No general cook at home recipes so had to add each individual ingredient annoying.

r14, female, 31-40 years old

I also wish it was easier to add meals I’ve made myself where I don’t know the nutritional value.

r16, female, 18-30 years old

This raised concerns around the inaccuracies and difficulty of matching foods consumed to those available in the database:

I do a lot of own cooking & recipes don’t have calorie count in them so hard to find exactly what you’re eating.

r10, female, 31-40 years old

It was suggested that features to share food items could be incorporated:

Being able to share food items with another person.

r13, female, 51-60 years old

Although 1 patient had indicated that they liked the app’s ability to take photos, they also offered a suggestion for improvement by allowing the app to:

Access to photos—the first few times I took photos on my phone and then wanted to upload them into easy diet diary, but I don’t think this is possible. You have to take the photos through easy diet diary [sic].

r12, female, 31-40 years old

#### Easy Diet Diary App Usability From Dietitians’ Perspectives

Dietitians also provided some input around the usability of Easy Diet Diary with their patients. The limited compatibility across both iOS and Android platforms was a practical constraint:

The only challenge I have had is that you suggest the Easy Diet App and then find out the patients has a Samsung.

Patient experience and familiarity with using apps and the age of patients were practical considerations for dietitians when prescribing the app to their patients, for example:

It was user friendly for people who were used to using apps.

I have older patients who are not well and they struggled to use it.

Other features dietitians suggested to be included or improved were:

an integrated exercise monitor—most patients ignored entering ex[ercise] as it was too complicated and difficult to enter accurately. Also to make it easier to enter personal recipes.

## Discussion

### Principal Findings and Comparison With Other Literature

To our knowledge, this is the first study to provide evidence for the feasibility of an intervention designed to train, educate, and provide opportunities for dietitians to improve their self-efficacy with using mHealth apps. Preliminary findings indicate that the workshop was effective in improving dietitians’ self-efficacy with using mHealth apps, with the effects maintained at 12 weeks. There were no apparent gains in patient satisfaction with nutrition care or dietetic services when prescribing an app to their patients. Both dietitians and their patients expressed willingness to continue using the connected platform and app. However, feedback on the inadequate time for administering the app during the consultation and the burden of logging meals and multi-ingredient recipes indicates that further investigation into streamlining app use in the nutrition care process is needed.

Marked improvements in dietitians’ self-efficacy with using mHealth apps were observed after attending the educational and skills training workshop component of this intervention. This is attributable to the workshop targeting the 4 sources of information proposed by Bandura that impact the development of individuals’ self-efficacy beliefs [7,9]. The workshop addressed barriers around dietitians’ lack of understanding about the best apps to use with patients and enabled them to acquire knowledge and familiarity around apps and to develop mastery of skills with using apps in various aspects of patient nutrition care. More importantly, the training allowed dietitians to build self-beliefs in their abilities to use mHealth apps through successful performance and practice of using the apps in a relaxed and engaging environment with expert facilitation and modeling and peer-to-peer support.

The progress made with dietitians’ self-efficacy toward using mHealth apps also aligns with understanding drawn from research into computer self-efficacy. Computer self-efficacy research models propose that antecedents of computer self-efficacy include prior performance experiences with using computers, computer knowledge, behavioral modeling in computer training, social support, and encouragement provided by similar others, such as colleagues [34-38], which coincide with the areas targeted by this workshop.

Personal accomplishments and successes with performing a task can raise individuals’ beliefs and expectations in their own capabilities [7,9]. The inclusion of a connected app platform in the 12-week phase provided further opportunities for mastery experiences. Dietitians had the opportunity to engage in additional mHealth-related tasks such as reviewing patient records to develop a stronger efficacy with implementing and integrating mHealth apps into their work systems. There was no significant increase in overall mHealth app self-efficacy ratings between postworkshop and 12-week measures. However, the maintenance rather than the decline of self-efficacy scores at 12 weeks is likely to be attributable to the sustained effort and increased frequency of prescribing apps to patients and using the connected app platform and the resilience to barriers and challenges [7].

When considering patient acceptance of using mHealth technologies in their chronic disease management, the relationship of a patient with their health practitioner can influence their perceived ease of use of an app [39]. As such, the implication is that when apps are prescribed by dietitians who have a good rapport with patients, this may reduce resistance to change and increase intention to use the app [39]. Furthermore, the accountability offered through dietitians reviewing their patients’ progress in the connected app platform was found to motivate patients to continue using the app. This is consistent with the supportive accountability model, which proposes that human support provided by a trustworthy expert coach, such as a dietitian, can enhance adherence to online behavior change interventions [40].

In the hospital setting, mHealth apps are indicated to improve patient experience, with 1 study determining that the use of mHealth apps during a hospital visit was able to increase the outpatient experience ratings by 17.7% [41]. However, such enhancements to patient satisfaction between intervention and control patients from the use of apps in patient care were not observed in this study. An explanation for the lack of significant improvements between groups may be the high satisfaction that control patients had toward impact, professionalism, and expectations toward dietetic care and services. This is consistent with the literature whereby patient satisfaction was higher with medical nutrition therapy for hypercholesterolemia delivered by dietitians than with the usual care offered by physicians [42]. Furthermore, these findings provide evidence that apps do not have any detrimental impact on patients’ perceptions over the quality of nutrition care.

The SUS score of above 70 achieved from patients’ assessments of Easy Diet Diary app indicates a *good* rating, suggesting that the app is an acceptable product [30,33]. However, the scores are comparatively lower than those of a quality assessment carried out by an expert dietetics app assessor, where it was ranked equal first with a perfect SUS score from among 28 popular nutrition weight loss apps [22]. In another study investigating a modified researcher version of the Easy Diet Diary app, the majority of participants also found the app easy to use and the barcode scanner to be useful [24]. However, only 52% of individuals agreed or strongly agreed that “the foods they usually eat were easy to find on the app” [24], which is comparable with the qualitative feedback of patients who found it difficult to match or locate their consumed food among all the choices.

Challenges relating to entering of home-cooked recipes have similarly been found in the researcher-version of the Easy Diet Diary app, where 64% of participants agreed that they often had to include their own recipes into the app [24]. It would be expected that as education and health behaviors change with dietetic intervention, there may subsequently be an increase in the frequency of meals cooked at home. Increased frequency of home-cooked meals is associated with improved diet quality and a greater likelihood of normal range body mass index and normal percentage body fat [43,44]. Therefore, a consideration for dietitians when prescribing apps in nutrition care is that over time as patients’ dietary habits change and there is more home cooking, it may become less convenient to use apps to log intake.

Although the usability of electronic health records (EHRs) by physicians is well studied [45], little is known about the usability of electronic platforms to support dietitians’ use of patient data from apps. The Easy Diet Diary Connect platform has a comparative SUS score to evaluations of certain EHRs [46]. However, other literature has highlighted that physicians perceive EHRs to have poor usability amidst a range of other limitations relating to inefficiencies from improper integration and interference with face-to-face patient care [45].

Country-specific mHealth apps and technology are valued by app users, patients, and dietitians alike [13,47] and present more accuracy for dietary assessment when used in the appropriate country’s context [24,47-49]. A specific *My Coach* function is available for Canadian dietitians to connect with patients using the Dietitians of Canada eaTracker app or website [21]. eaTracker provides the opportunity for a greater degree of nutrition care tailoring through personalized goal setting rather than general caloric intake targets [50,51]. myPace is a European dietitian-researcher–developed platform containing 3 interfaces (dietitian Web interface, patient mobile, or Web interface) and designed specifically to support the dietitian-patient relationship for sustainable weight loss and weight management [18,19]. The myPace platform allows dietitians to directly send motivational messages to their patients, a feature that dietitians felt was missing from the Easy Diet Diary Connect platform.

### Future Directions and Strengths and Limitations

The low response rate to this study and the small sample size of dietitians and patients is a clear limitation to the interpretation of results. This study was not powered for statistical analysis. However, the finding of some significant result of improved mHealth app self-efficacy for dietitians from such a small sample provides indication of its potential efficacy if a larger sample was to be obtained. As the study only recruited a small number of private practice dietitians, future dissemination of this intervention could offer the educational and skills training workshop outside business hours and across different locations, to allow for more private practice dietitians to attend.

A possible source of bias is that the dietitians who volunteered to participate in this study had a greater interest in engaging with technology and, thereby, were likely to have higher motivation for developing self-efficacy with using mHealth app technologies. Furthermore, this study only recruited dietitians who were not regular users of apps in their practice. With some redesigning of workshop content to offer education and training on more specific and advanced skills, it is likely to also be beneficial for increasing the mHealth app self-efficacy of existing app users, given that prior experience with using apps can predict stronger self-efficacy [36].

There is also translation potential of delivering such an intervention to doctors, nurses, and other health professional groups who, like dietitians [4], are commonly using apps in their own clinical practice [52,53]. The older and more experienced medical and health professionals would likely benefit more as junior doctors seem to be adopting apps already [54,55]. Overall, less than 10% of doctors had recommended mHealth apps to their patients [52]. The key barriers to not recommending apps align with those expressed by dietitians [4], including that the doctors had never thought about recommending them, followed by uncertainties over the evidence base, safety, the best apps to recommend, and efficacy of apps [52], all of which could be addressed through education and training.

From this study, short-term benefits of the educational and skills training workshop and integration of the connected platform were observed on dietitians’ self-efficacy with using mHealth apps in their practice. However, it would be necessary to conduct an extended study to examine how dietitians’ self-efficacy with using apps and their app use within the practice are sustained. Long-term changes to patient satisfaction following implementation apps into patient care should also be measured. In this study, parameters on the effectiveness of the intervention on patient outcomes were only provided through the mHealth app self-efficacy tool. It would be pertinent to examine the impact of the intervention on patient biochemical and anthropometric outcomes directly in future studies.

### Conclusions

This study has demonstrated the feasibility of improving dietitians’ self-efficacy with using mHealth apps in their practice through the implementation of an intervention that provided dietitians with education and skills-based training to develop capability, motivation, and mastery of performance with using apps. Determining further strategies to improve the integration of app platforms into the various patient health management systems used by dietitians and other health and medical practitioners could provide further opportunities for health professionals to build mHealth app self-efficacy so that the benefits of apps in health care service delivery can be realized. The qualitative findings of this study have provided a rich source of information on the usability of the app platform and the associated app in dietetic practice and patient care, and the suggested improvements should be considered by app developers. However, being a feasibility study in nature, further translational research is required to determine the impact of the intervention on long-term mHealth self-efficacy for the broader dietetic profession and for patient outcomes.

## Abbreviations

ANOVA: analysis of variance
APD: Accredited Practising Dietitian
COM-B: Capability, Opportunity, Motivation-Behavior
EHR: electronic health records
mHealth: mobile health
SUS: system usability scale
